# Author Correction: Hypomorphic *Brca2* and *Rad51c* double mutant mice display Fanconi anemia, cancer and polygenic replication stress

**DOI:** 10.1038/s41467-024-46561-9

**Published:** 2024-03-12

**Authors:** Karl-Heinz Tomaszowski, Sunetra Roy, Carolina Guerrero, Poojan Shukla, Caezaan Keshvani, Yue Chen, Martina Ott, Xiaogang Wu, Jianhua Zhang, Courtney D. DiNardo, Detlev Schindler, Katharina Schlacher

**Affiliations:** 1 Department of Cancer Biology, The University of Texas MD Anderson Cancer Center, Houston, TX 77054 USA; 2 Department of Neurosurgery, The University of Texas MD Anderson Cancer Center, Houston, TX 77054 USA; 3 Department of Genomic Medicine, The University of Texas MD Anderson Cancer Center, Houston, TX 77054 USA; 4 Department of Leukemia, The University of Texas MD Anderson Cancer Center, Houston, TX 77054 USA; 5 Institut fuer Humangenetik, University of Wuerzburg, Wuerzburg, Germany

**Keywords:** Cancer, Genetics

Correction to: *Nature Communications* 10.1038/s41467-023-36933-y, published online 11 March 2023

In the original version of the published article, the Acknowledgements section was missing a statement about the funding source for two graduate students: Carolina Guerrero and Poojan Shukla were supported by the CPRIT Research Training Award CPRIT Training Program (RP210028).

The correct Acknowledgements paragraph is:

We thank Drs. John A. Tainer, Davide Moiani, Michael Longo and Gareth Williams for sharing information and discussions on the RAD51C crystal structure, Dr. Tamara M. Haygood for advice on tail pathologies seen with X-ray, as well as the MDACC Genetically Engineered Mouse Facility (GEMF), the Small Animal Imaging Facility, the Research Histology Core Laboratory core facilities, the Advanced Cytometry & Sorting Facility at South Campus (ACSF), and the Veterinary and Comparative Pathology facility at MD Anderson Cancer Center for critical support. The work was supported by the NIEHS under award 1R01ES029680, and by CPRIT RP180463, R1312 and RP180813 (K.S.), and the FA research group at the University of Wuerzburg was supported by grants from the Schroeder Kurth Fund (D.S.). Carolina Guerrero and Poojan Shukla were supported by the CPRIT Research Training Award CPRIT Training Program (RP210028). K.S. is a Rita Allen Foundation Fellow and a CPRIT scholar in Cancer Biology (previous award R1312).

This has now been corrected both in the HTML and the PDF versions of the article.

