# Supplementary material for: Hypomorphic Brca2 and Rad51c double mutant mice display Fanconi anemia, cancer and polygenic replication stress
Source: Nat Commun. 2023 Mar 11;14:1333. doi: 10.1038/s41467-023-36933-y (PMC10008622; doi:10.1038/s41467-023-36933-y)
Supplement: Supplementary file 1 — Supplementary Information [file 41467_2023_36933_MOESM1_ESM.pdf]

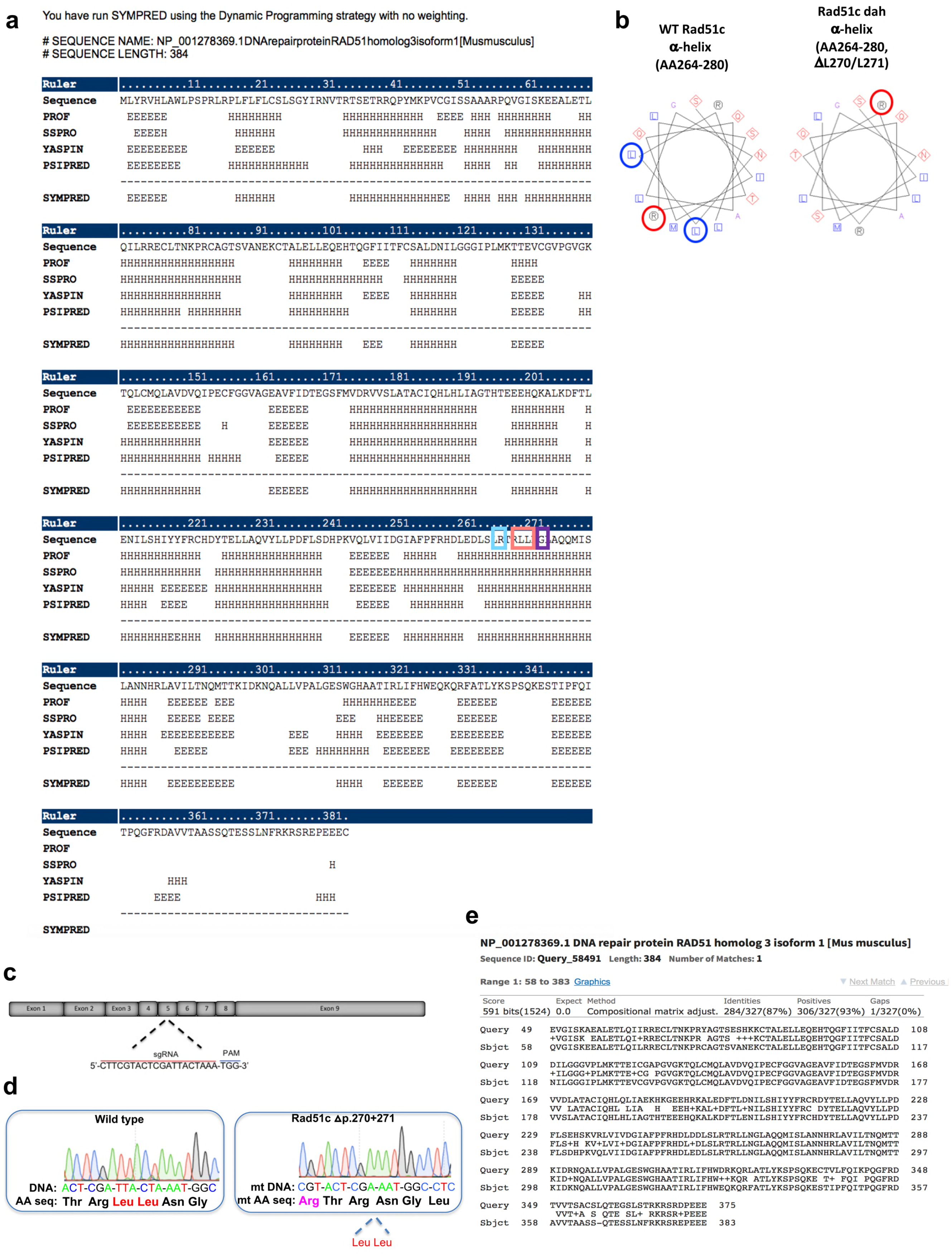

Supplementary Fig.1. Generation of Rad51c mutant mice with 6bp deletion.

a, Schematic diagram of sgRNA guide and targeting sites in the mouse *Rad51c* locus.
PAM = protospacer adjacent motif.
b, wheel projection of alpha helix amino acids 264-280 of murine Rad51c (left), and wheel projection of the same alpha helix in the Rad51c dah mutant missing L270/L271.
c, Sanger sequencing of PCR products encompassing the *Rad51c* targeting region from a representative wild-type (WT) mice and *Rad51c*$^{dah/dah}$ mouse. The two leucine residues that are deleted in *Rad51c*$^{dah/dah}$ mouse are marked in red, the adjacent Arginine that is substituted by a histidine in the FANCO patient is marked in magenta.
d, Blast sequence alignment of human (NP_478123.1) and murine RAD51C (NP_001278369) shows high similarity and evolutionary conservation.
e, SymPRED protein secondary structure prediction of RAD51C showing R258 (blue square, mutated to histidine in the FANCO patient) adjacent to the two leucines (red square) that are deleted in the mouse model within the same a-helix

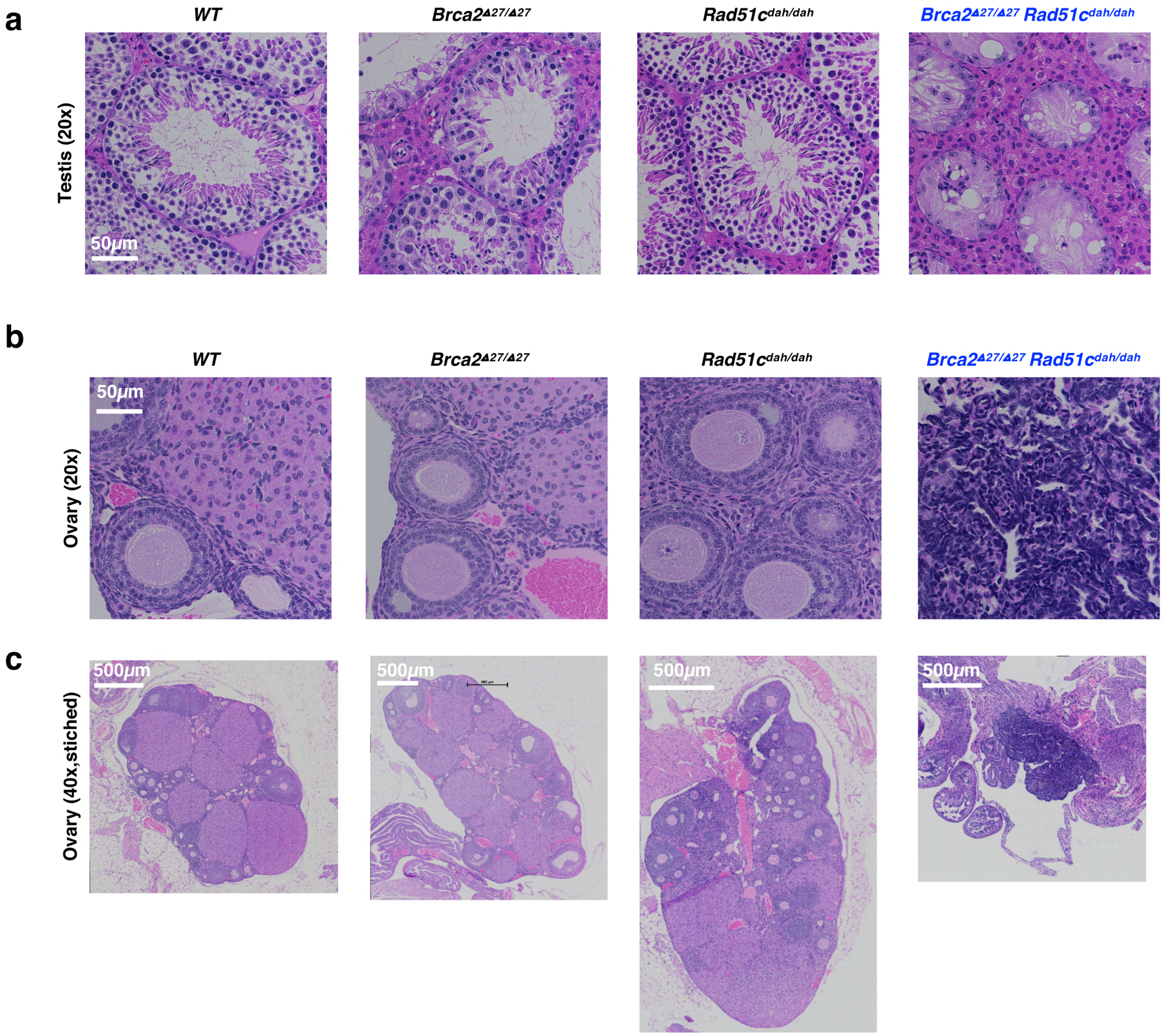

Supplementary Fig.2. Polygenic mutant FANC mice show impaired gonad development.
a, Representative images of hematoxylin and eosin (H&E)-stained tissue cross-sections of testis from animals with indicated genotypes at 8-12 weeks. Seminiferous tubuli of *Brca2*$^{Δ27/Δ27}$ mice can show decreased spermatocytes (arrow), while those of *Brca2*$^{Δ27/Δ27}$ +*Rad51c*$^{dah/dah}$ mice show a Sertoli cell only phenotype with a complete loss in spermatocytes and spermatogonia. Scale bars indicate 50µm. Similar results were obtained by analyzing three independent tissues of each genotype.
b-c, Representative images of H&E-stained tissue cross-sections of ovaries from animals with indicated genotypes at 12 weeks. Scale bars indicate 50mm **b,** and 500µm **c**. Similar results were obtained by analyzing three independent tissues of each genotype.

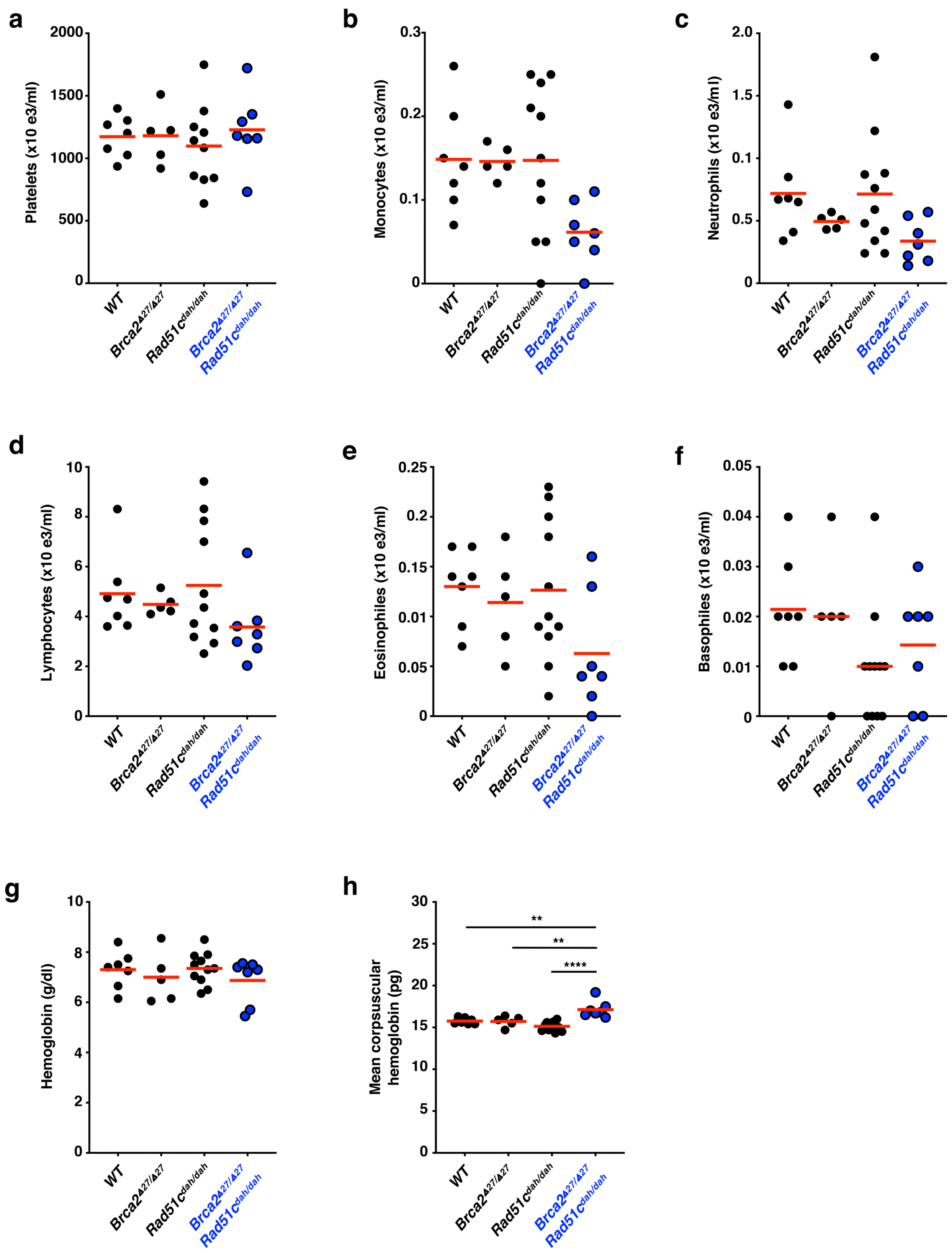

Supplementary Fig.3. Complete blood count analysis of *Brca2$^{\Delta27/\Delta27}$* +*Rad51c$^{dah/dah}$* mice.

a-h, Complete blood count analysis of 8–12 weeks old *Brca2*$^{\Delta 27/\Delta 27}$ +*Rad51c*$^{dah/dah}$ and control mice. Data represent biologically independent samples for wild-type (n=7), *Brca2*$^{\Delta 27/\Delta 27}$(n=5), *Rad51c*$^{dah/dah}$ (n=11, except a, platelet count only contain n=10) and *Brca2*$^{\Delta 27/\Delta 27}$+*Rad51c*$^{dah/dah}$ (n=7) mice.
a, Platelet count.
b, Monocyte count.
c, Neutrophils count.
d, Lymphocytes count.
e, Eosinophil count.
f, Basophil count.
g, Hemoglobin concentrations.
h, Mean corpuscular hemoglobin concentration. *Brca2*$^{\Delta 27/\Delta 27}$+*Rad51c*$^{dah/dah}$ against WT: p=0.0026; *Brca2*$^{\Delta 27/\Delta 27}$+*Rad51c*$^{dah/dah}$ against *Brca2*$^{\Delta 27/\Delta 27}$: p=0.0053; ; *Brca2*$^{\Delta 27/\Delta 27}$+*Rad51c*$^{dah/dah}$ against *Rad51c*$^{dah/dah}$: p<0.0001.
Red bar in scatter graphs represent the mean. *p*-values are derived using the one-way ANOVA test. ** $p < 0.01$, **** $p < 0.0001$.

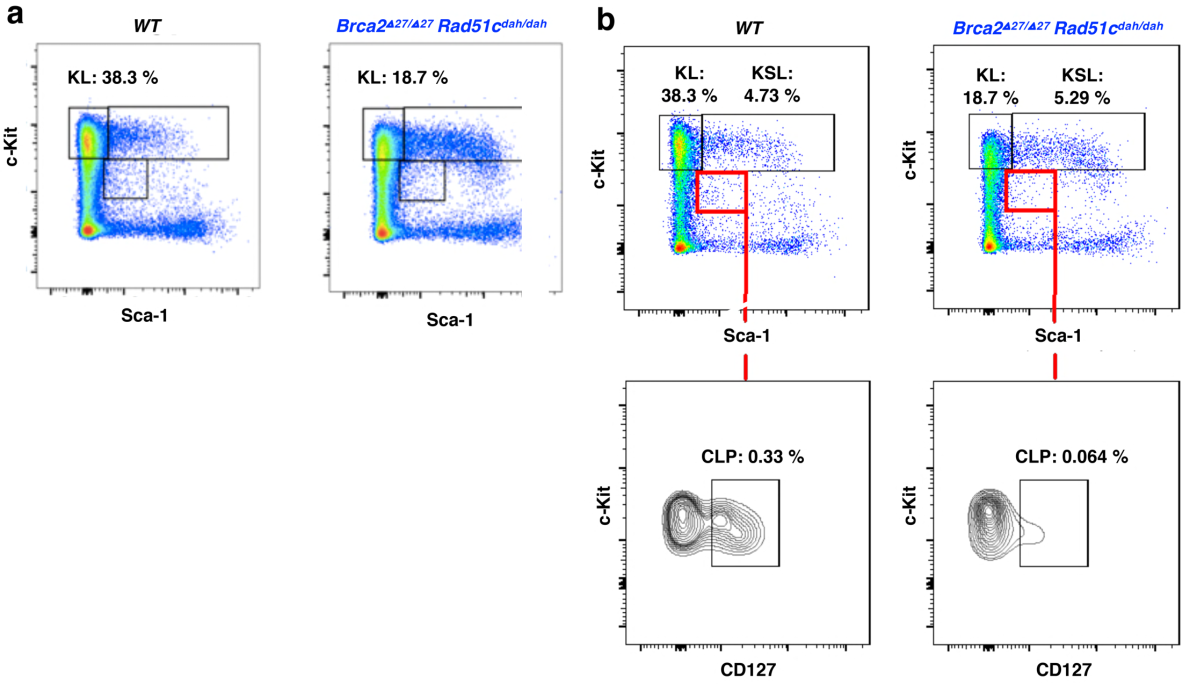

Supplementary Fig.4. Hematological FACS analysis of *Brca2*$^{\Delta 27/\Delta 27}$ +*Rad51c*$^{dah/dah}$ mice.

a, Representative FACS plot showing gating for hematological lineage analysis performed for bone marrow cells collected from 8-12 weeks old mice with indicated genotypes (n=5-6). Lineage negative cells (20,000 events) are gated for CD117 (c-Kit) positive and Sca-1 negative populations to identify the KL subset designating myeloid progenitor cells.

b, Representative FACS plot showing gating for identification of the CLP subset, designating common lymphoid progenitors. Lineage negative cells (20,000 events) are gated for c-Kit positive and Sca-1 moderate expression (KSL), which is further gated for CD127 positive cells to identify the CLP subpopulation designating the lymphoid population.

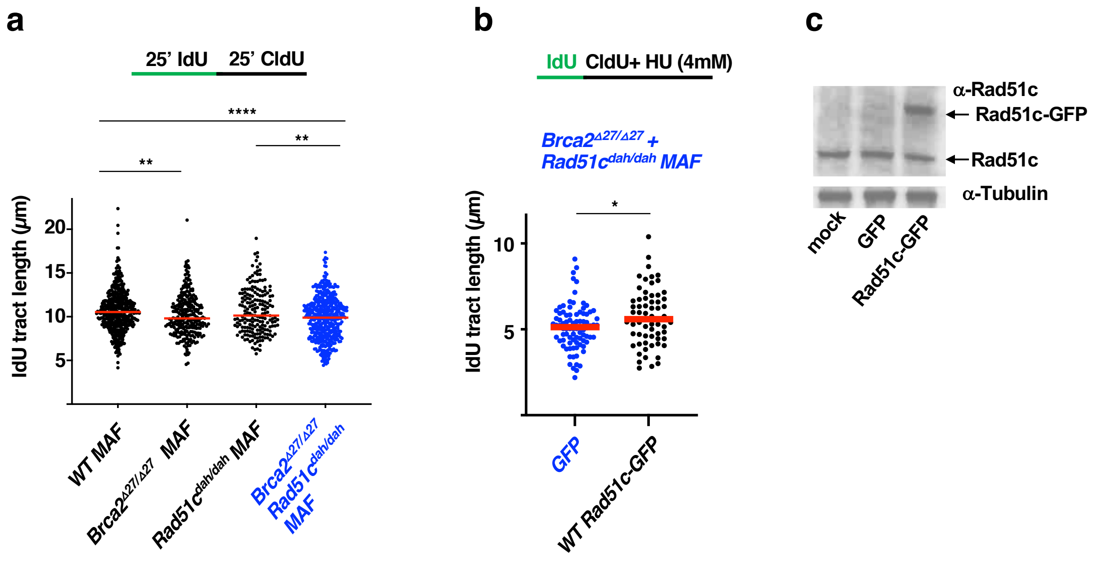

Extended Data Fig.5. Cells with polygenic *FANC* mutations show reduced DNA replication speeds.
a, Scatter plot of DNA fiber analysis of nascent IdU tract lengths incorporated without external replication stalling, measuring DNA replication progression. Data represent biologically independent fiber experiments from wild-type (n=5), $Brca2^{\Delta27/\Delta27}$(n=4), $Rad51c^{dah/dah}$ (n=2) and $Brca2^{\Delta27/\Delta27}$+$Rad51c^{dah/dah}$ (n=4) mouse embryonic fibroblasts (MAF). In total, analysis was performed for wild-type (n_{fibers}=469), $Brca2^{\Delta27/\Delta27}$($n_{fibers}$ =270), $Rad51c^{dah/dah}$ (n_{fibers} =190) and $Brca2^{\Delta27/\Delta27}$+$Rad51c^{dah/dah}$ (n_{fibers} =462) fibers. $Brca2^{\Delta27/\Delta27}$ against WT : $p = 0.0019$, $Brca2^{\Delta27/\Delta27}$+$Rad51c^{dah/dah}$ against WT : $p < 0.0001$ and $Rad51c^{dah/dah}$ against $Brca2^{\Delta27/\Delta27}$+$Rad51c^{dah/dah}$: $p = 0.0023$.
b, Scatter plot of DNA fiber analysis of nascent IdU tract lengths before 4mM hydroxyurea in $Brca2^{\Delta27/\Delta27}$+$Rad51c^{dah/dah}$ MAFs transfected with empty GFP vector or wild-type Rad51c-GFP. Experiment was performed twice and representative fiber analysis for MAFs transfected with empty GFP vector (n=86) or wild-type Rad51c-GFP (n=64) with $p = 0.0244$ between the two groups.
c, Western blot of mock transfected, empty GFP vector transfected, and wild-type Rad51c-GFP transfected $Brca2^{\Delta27/\Delta27}$+$Rad51c^{dah/dah}$ MAFs. Experiment was performed once.
p-values for DNA fiber analysis are derived using the two-tailed Mann-Whitney test, * $p < 0.05$, *** $p < 0.001$, **** $p < 0.0001$, n.s. $p > 0.05$.

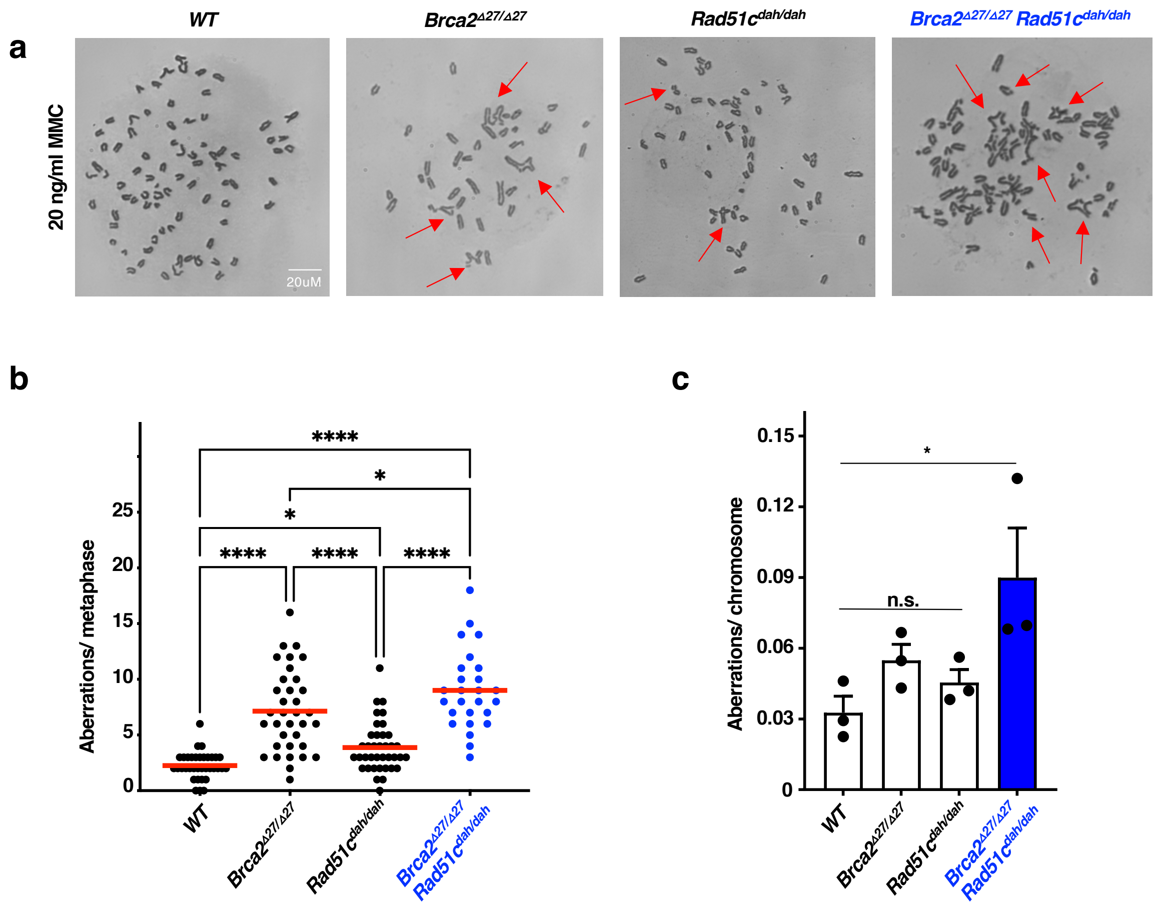

Supplementary Fig.6. Cells with polygenic FANC mutations show increased genomic aberrations.
a, Representative images of metaphase chromosome spreads in mouse adult fibroblasts (MAF) of indicated genotypes with 20 ng/ml mitomycin C (MMC). Examples for chromosomal aberrations are indicated with an arrow.
b, Scatter dot blot of chromosomal aberrations in metaphase spreads of MAFs after treatment with 20ng/ml MMC as indicated. Data represent 2 biologically independent experiments. In total, chromosome analysis was performed for 20ng/ml MMC treated wild-type (n=35), $Brca2^{\Delta 27/\Delta 27}$(n=35), $Rad51c^{dah/dah}$ (n=38) and $Brca2^{\Delta 27/\Delta 27}$+$Rad51c^{dah/dah}$ (n=25) cells. $Brca2^{\Delta 27/\Delta 27}$+$Rad51c^{dah/dah}$ against WT and $Rad51c^{dah/dah}$: p<0.0001; $Brca2^{\Delta 27/\Delta 27}$+$Rad51c^{dah/dah}$ against $Brca2^{\Delta 27/\Delta 2}$: p=0.0268; $Brca2^{\Delta 27/\Delta 27}$ against WT and $Rad51c^{dah/dah}$: p<0.0001; $Rad51c^{dah/dah}$ against WT: p=0.0403
c, Bar graph of spontaneous chromosome aberrations per chromosome (without MMC) in metaphase spreads in MAFs. Data represent the average aberrations/chromosome of three biologically independent experiments. In total, analysis was performed for wild-type (n=50), $Brca2^{\Delta 27/\Delta 27}$(n=52), $Rad51c^{dah/dah}$ (n=46) and $Brca2^{\Delta 27/\Delta 27}$+$Rad51c^{dah/dah}$ (n=53) cells. $Brca2^{\Delta 27/\Delta 27}$+$Rad51c^{dah/dah}$ against WT: p=0.0379. Error bars represent the standard error of the mean. The data represent the mean of compiled data from biological repeats. p-values are derived using the one-way ANOVA test. * $p < 0.05$, **$p < 0.01$, *** $p < 0.001$, **** $p < 0.0001$

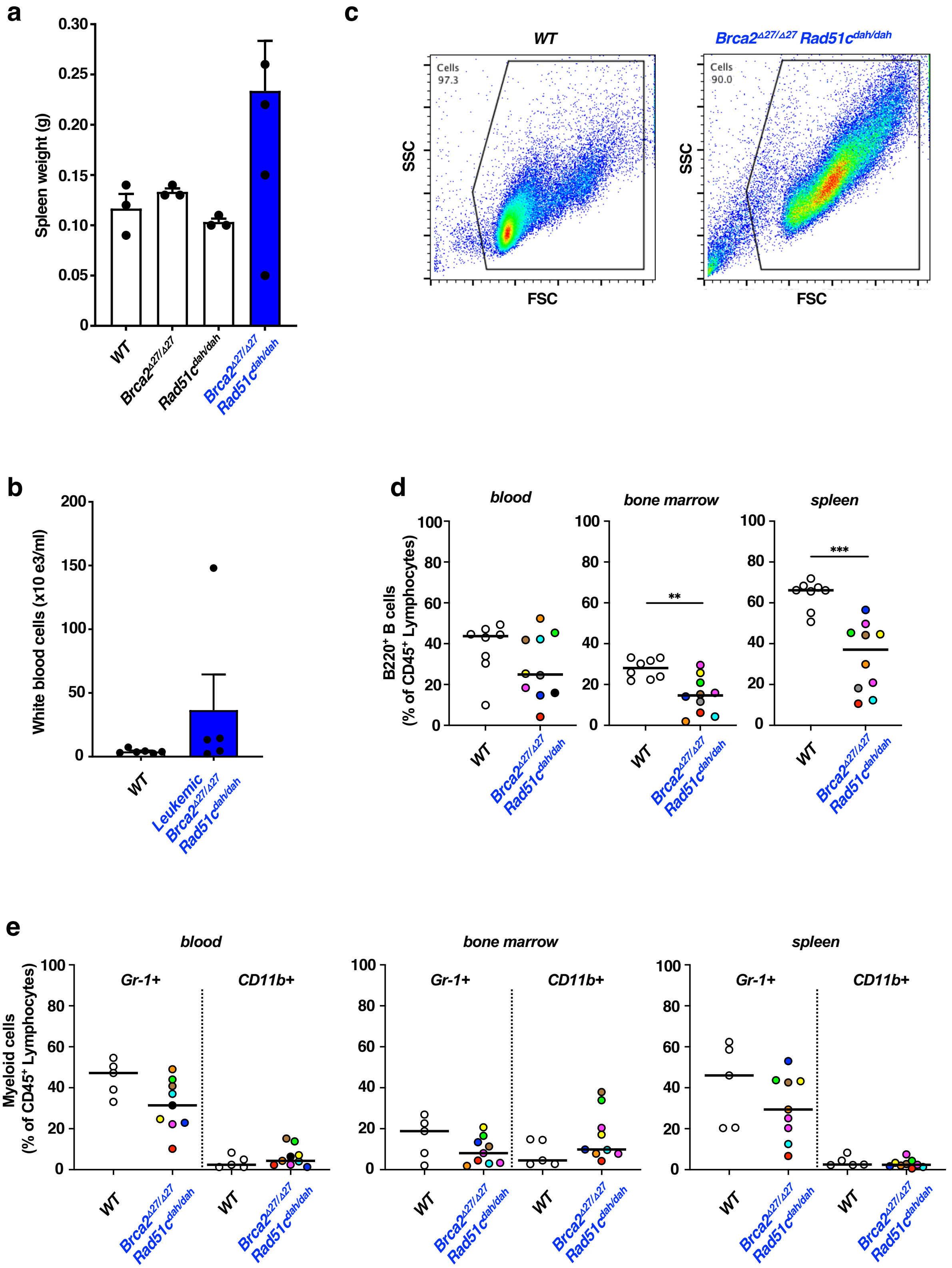

a
Spleen weight (g)
0.30
0.25
0.20
0.15
0.10
0.05
0
WT
Brca2Δ27/Δ27
Rad51cdah/dah
Brca2Δ27/Δ27 Rad51cdah/dah
b
White blood cells (x10 e3/ml)
200
150
100
50
0
WT
Leukemic Brca2Δ27/Δ27 Rad51cdah/dah
c
WT
Brca2Δ27/Δ27 Rad51cdah/dah
Cells 97.3
Cells 90.0
SSC
FSC
d
blood
bone marrow
spleen
B220+ B cells (% of CD45+ Lymphocytes)
100
80
60
40
20
0
**
***
WT
Brca2Δ27/Δ27 Rad51cdah/dah
e
blood
bone marrow
spleen
Myeloid cells (% of CD45+ Lymphocytes)
Gr-1+
CD11b+
100
80
60
40
20
0
WT
Brca2Δ27/Δ27 Rad51cdah/dah

Supplementary Fig.7. ***Brca2**$^{\Delta27/\Delta27}$* ***+Rad51c**$^{dah/dah}$* **mice show abnormalities in tissues of the immune system.**

a, Bar-graph of enlarged spleen quantification in malade *Brca2*$^{\Delta27/\Delta27}$ *+Rad51c*$^{dah/dah}$ and age-matched control mice. Data represent biologically independent samples for wild-type (n=3), *Brca2*$^{\Delta27/\Delta27}$ (n=3), *Rad51c*$^{dah/dah}$ (n=3) and *Brca2*$^{\Delta27/\Delta27}$*+Rad51c*$^{dah/dah}$ (n=5) mice.

b, Representative forward and side-ward scatter FACS plot image of thymus cells from WT and malade *Brca2*$^{\Delta27/\Delta27}$ *+Rad51c*$^{dah/dah}$ mice show enlargement of the mutant cells.

c, White blood cell count of leukemic *Brca2*$^{\Delta27/\Delta27}$ *+Rad51c*$^{dah/dah}$ mice shows increased counts compared to age-matched wild-type (WT) mice. Data represent biologically independent blood samples from wild-type (n=7) and leukemic *Brca2*$^{\Delta27/\Delta27}$*+Rad51c*$^{dah/dah}$ (n=5) mice.

Error bars represent the standard error of the mean.

d, e, Flow cytometry analysis of blood, bone marrow and spleen cells from malade *Brca2*$^{\Delta27/\Delta27}$*+Rad51c*$^{dah/dah}$ mice identifying B-Cells (**d**, B220+) or a myeloide population (**e**, Gr-1 or CD11b) as percentage of total lymphocytes (CD45+). Data represent biologically independent samples for wild-type (n=8) and *Brca2*$^{\Delta27/\Delta27}$*+Rad51c*$^{dah/dah}$ (n=10) mice for identifying B-Cells (**d**, B220+). Data represent biologically independent samples for wild-type (n=5) and *Brca2*$^{\Delta27/\Delta27}$*+Rad51c*$^{dah/dah}$ (n=9) mice for identifying myeloide populations (**e**, Gr-1 or CD11b). *Brca2*$^{\Delta27/\Delta27}$*+Rad51c*$^{dah/dah}$ against WT for bone marrow: p=0.0021; and for spleen p=0.0002.

p-values for flow cytometry analysis are derived using the two-tailed Mann-Whitney test. * $p < 0.1$, **$p < 0.01$, *** $p < 0.001$, **** $p < 0.0001$.

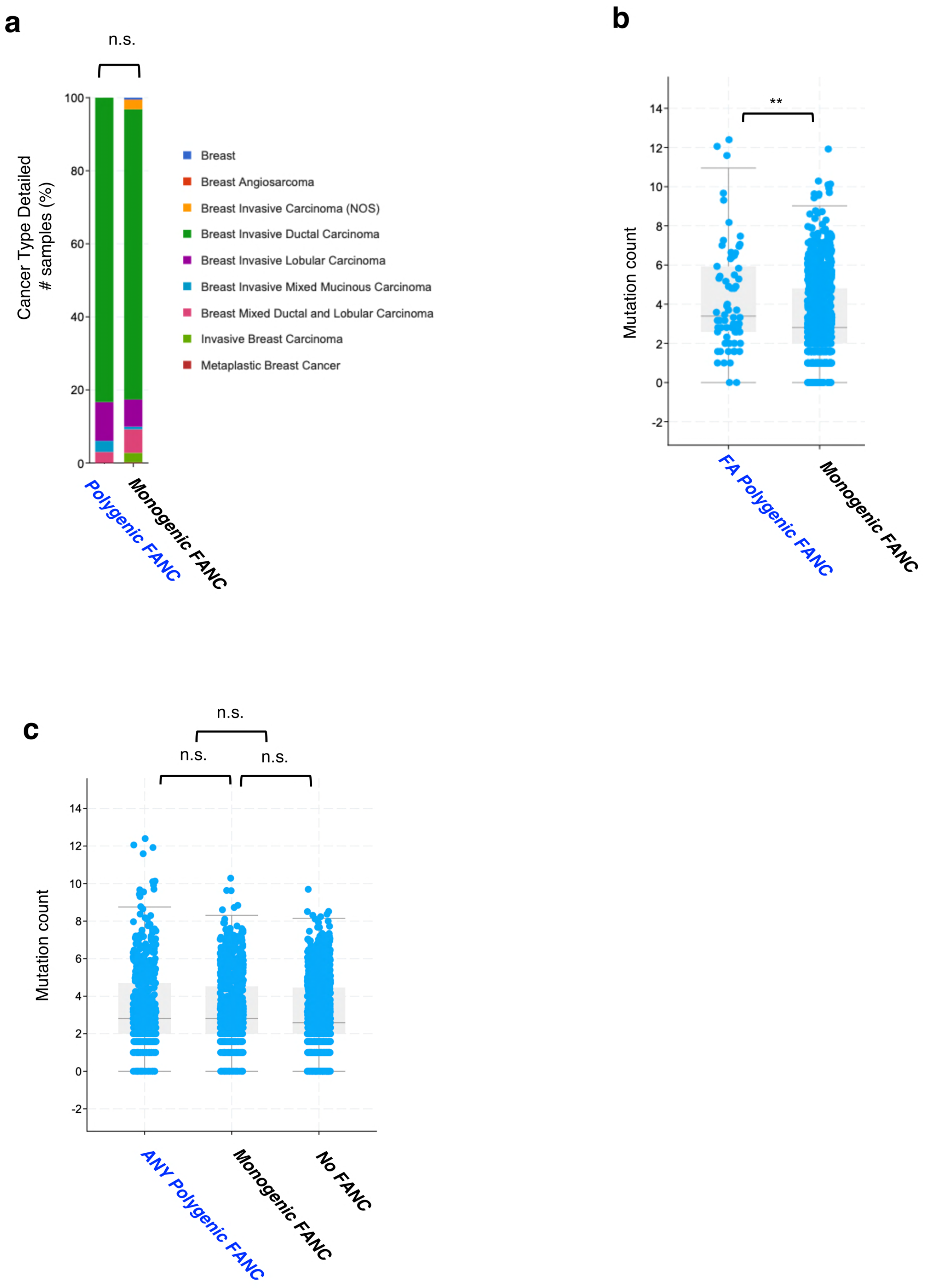

Supplementary Fig.8. Breast cancer patients with polygenic FANC tumor mutations show increased mutagenic count.

a, Bar-graph of cancer types of patients with polygenic and monogenic FANC tumor mutations (*p*=0.262). *p*-values were derived using the two-sided Chi-squared test.
b, Scatter plot of mutation counts of tumor samples from 3593 patients with polygenic and monogenic FANC tumor mutations, whereby only polygenic FANC mutation combinations were considered that are found in FA patients. Box centres indicate median, boundaries represent 25th and 75th percentiles, and error bars represent maximum and minimum values. *p*-values were derived using the two-sided Kruskal-Wallis test, **p=0.014.
c, Scatter plot of mutation counts of tumor samples from 3593 patients with polygenic and monogenic FANC tumor mutations, whereby any combination of two FANC tumor mutations in the 23 FANC genes were considered as polygenic FANC mutation. Box centres indicate median, boundaries represent 25th and 75th percentiles, and error bars represent maximum and minimum values. *p*-values were derived using the two-sided Kruskal-Wallis test.

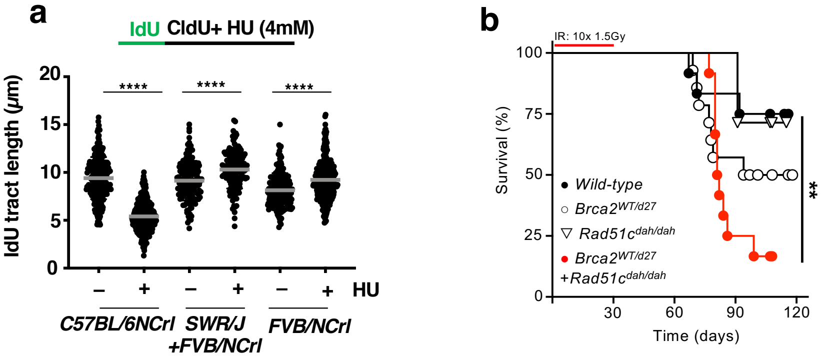

Supplementary Fig.9. Mouse strain backgrounds vary in intrinsic fork protection defects.
a, Scatter plot of DNA fiber analysis of nascent IdU tract lengths incorporated before replication stalling with high concentrations of hydroxyurea (HU, 4mM) and without, measuring DNA fork protection as the reduction of IdU tract lengths with HU in MAFs in various mouse strain backgrounds as indicated. Data represent two independent experiments. In total, fiber analysis was performed for untreated (n=245) and HU treated (282) C57BL/6NCrl MAFs, untreated (n=140) and HU treated (148) SWR/J+ FVB/NCrl mixed background MAFs and untreated (n=189) and HU treated (n=310) FVB/NCrl MAFs. *p*-values for DNA fiber analysis are derived using the two-tailed Mann-Whitney test, **** $p < 0.0001$ for all comparison of untreated and treated samples in each group.
b, Kaplan-Meier curves for overall survival of male wild-type (WT), *Brca2*$^{WT/\Delta27}$, *Rad51c*$^{dah/dah}$, and *Brca2*$^{WT/\Delta27}$*+Rad51c*$^{dah/dah}$ mice after ten irradiations with 1.5 Gy daily. *Brca2*$^{WT/\Delta27}$*+Rad51c*$^{dah/dah}$ against WT: p=0.0091. *p*-values for comparing survival of two groups are derived using the Mantel-Cox test,

Uncropped Western blots of Supplementary Fig. 5c.
Anti-Rad51c blot: lane 1: protein marker, lane 2: mock transfected, lane 3: mock-GFP transfected, lane 4: wild-type Rad51c-GFP transfected *Brca2*$^{\Delta27/\Delta27}$*+Rad51c*$^{dah/dah}$ MAFs. Higher exposure blot to the right.

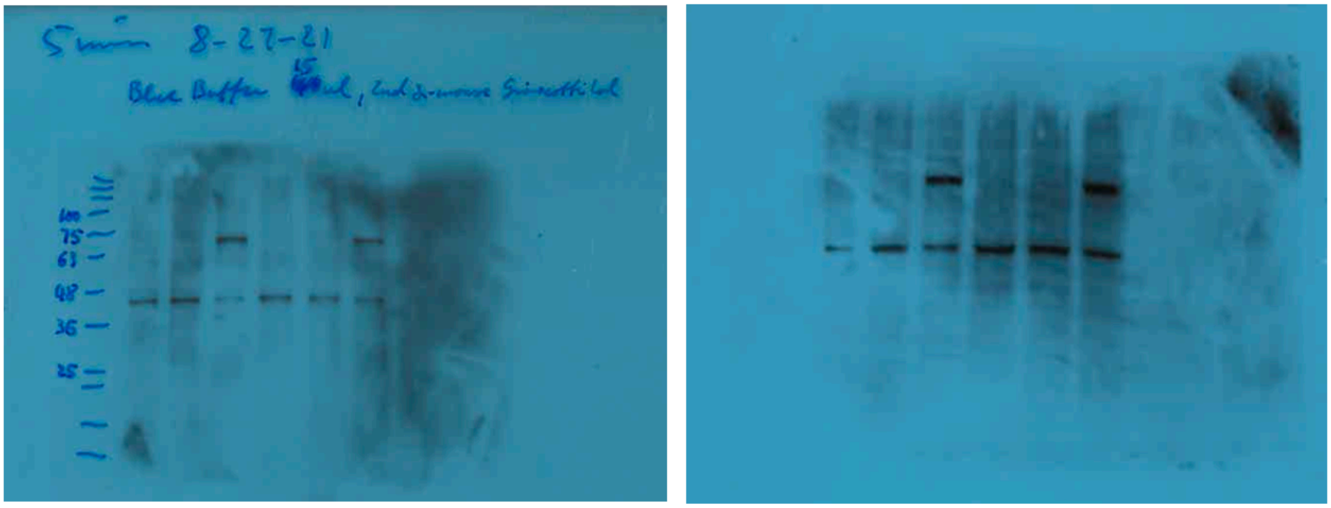

Anti-a-tubulin blot: lane 1: protein marker, lane 2: mock transfected, lane 3: mock-GFP transfected, lane 4: wild-type Rad51c-GFP transfected *Brca2*$^{\Delta 27/\Delta 27}$+*Rad51c*$^{dah/dah}$ MAFs.

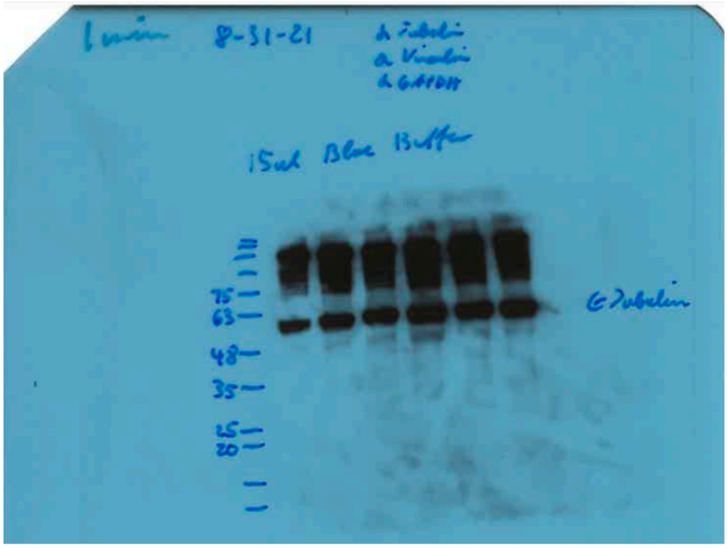

Supplementary Tables and References

ID	FA Subtype	Additional FANC mutations	Reference
HSC230	FA-B	‡*BRCA2/FANCD1* c.2805_2808delAAAC, p.(Ala938Profs*21)	(*1*)
EUFA867	FA-A	‡*FANCM* c.2171C>A, p.(Ser724*);<br>*FANCM* c.4222+1978_4300del, p.(fs*) [EX15del]	(*2*), (*3*)
FA4	FA-C	*BRCA2/FANCD1* c.951A>G, p.(N317S)	(*4*)
FA26	FA-L	*FANCJ* c.3737C>T, p.(P1246L)	(*4*)
Fa-004	FA-D2	*BRCA2/FANCD1* chr13:31809499C>G (NCBI36/hg18), p.(H1003D)	(*5*)
Fa-001	FA-A	*FANCI* chr15:87636941A > G, missense I > V	(*5*)
Fa-002	FA-A	‡*FANCB* chrX:14781120C > T, missense V > I;<br>‡*BRCA2* chr13:31871012A > C, 3'-UTR	(*5*)
Fa-003	FA-M	‡*BRCA2* chr13:31804480A > C, missense N > H	(*5*)

Supplementary Table 1. Previously reported *FANC* passenger mutations. Literature search of previously reported FA patients with germline *FANC* gene mutations in addition to a biallelic or hemizygous *FANC* inactivation. ‡ Denotes homozygous or biallelic additional *FANC* mutation.

Ser.#	Subtype	*FANC* passenger Mutation (non-synonymous)
**1**	FA-A	*FANCG* c.418C>T, p.(His140Tyr)
**2**	FA-F	*BRCA2/FANCD1* c.3854del, p.(Asn1287Ilefs*6)
**3**	FA-C	none
**4**	FA-A	none
**5**	FA-N	none
**6**	FA-C	none
**7**	FA-C	none
**8**	FA-A	none
**9**	FA-A	none
**10**	FA-A	none
**11**	FA-A	none
**12**	FA-N	none
**13**	FA-A	none
**14**	FA-G	none
**15**	FA-A	none
**16**	FA-A	none
**17**	FA-A	*SLX4/FANCP* c.734C>T, p.Pro245Leu,
**18**	FA-A	none
**19**	FA-A	*BRCA1/FANCS* c.301+7G>A
**20**	FA-Q	none
**21**	FA-A	none
**22**	FA-A	none
**23**	FA-A	none
**24**	FA-A	none
**25**	FA-A	none
**26**	FA-A	none
**27**	FA-D2	none
**28**	FA-C	none
**29**	FA-A	*FANCG* c.77A>G, p.Gln26Arg;<br>*FANCI* c.1305G>A, p.(Met435Ile)
**30**	FA-G	none
**31**	FA-M	*FANCA* c.2151G>T, p.(Met717Ile);<br>*RAD51C/FANCO* c.29T>G, p.(Met10Arg)
**32**	FA-B	*BRCA2/FANCD1* c.6725A>G, p.(Asp2242Gly);<br>*BRCA2/FANCD1* c.9104A>C, p.(Tyr3035Ser)
**33**	FA-B	none
**34**	FA-A	*SLX4/FANCP* c.3334A>C, p.(Lys1112Gln)
**35**	FA-D2	none
**36**	FA-A	*FANCE* c.1099C>A, p.(Leu367Ile)

Supplementary Table 2. *FANC* passenger mutations in whole-exome sequences of 36 FA patients. Whole exome sequencing of FA patient DNAs with previously diagnosed biallelic or hemizygous *FANC* inactivation (subtype) was performed and filtered for additional heterozygous non-synonymous, ACMG class 3 or higher variants in any one of the so far identified 23 *FANC* genes. Only exomes that could be re-evaluated unambiguously were used.

**Phenotype**	***BRCA2* mutation**	***RAD51C* mutation**	**Reference**
***Fanconi Anemia**	Hom. BRCA2 V2466A Het. BRCA2 N372H	Het. RAD51C G264S	(*6*) (*7*) (*8*)
**Breast cancer susceptibility**	Het. BRCA2 Δex26	Het. RAD51C R258H	(*9*) (*10*)

Supplementary Table 3. Polygenic *BRCA2+RAD51C* mutations are found in a Fanconi Anemia patient and families with a history of breast cancer. BRCA2 V2466A destabilizes the protein, BRCA2 N372H is a low-penetrance variant significantly associated with an increased risk of overall cancer , RAD51C G264S is a deleterious mutation predicted by SIFT and Polygen, BRCA2 Δex26 (L3216L) causes C-terminal internal deletions of exon 26, RAD51C R258H is a damaging mutation found in FA and breast cancer patients.
* The data/analyses presented in the current publication are based on the use of study data downloaded from the dbGaP web site, under phs001481.v1.p1, https://www.ncbi.nlm.nih.gov/projects/gap/cgi-bin/study.cgi?study_id=phs001481.v1.p1

a

**Parental genotype**		**Offspring genotype**				
**Father**	**Mother**	***Brca2***<br>***Rad51c***	***+/+***<br>***dah/dah***	***+/d27***<br>***dah/dah***	***d27/d27***<br>***dah/dah***	**Statistical Significance**
Brca2*$^{d27/+}$**<br>Rad51c*$^{dah/dah}$**	***Brca2*$^{d27/+}$**<br>***Rad51c*$^{dah/dah}$**	**Expected frequency**	**25.00%**	**50.00%**	**25.00%**	**p=0.0001, n=132**
		**Observed frequency**	**32.58%**	**56.82%**	**10.61%**	

b

**Parental genotype**		**Offspring genotype**										
**Father**	**Mother**	***Brca2***<br>***Rad51c***	***+/+***<br>***+/+***	***+/+***<br>***+/dah***	***+/+***<br>***dah/dah***	***+/d27***<br>***+/+***	***+/d27***<br>***+/dah***	***+/d27***<br>***dah/dah***	***d27/d27***<br>***+/+***	***d27/d27***<br>***+/dah***	***d27/d27***<br>***dah/dah***	**Statistical Significance**
Brca2*$^{d27/+}$**<br>Rad51c*$^{dah/+}$**	***Brca2*$^{d27/+}$**<br>***Rad51c*$^{dah/+}$**	**Expected frequency**	**6.25%**	**12.5%**	**6.25%**	**12.5%**	**25.00%**	**12.5%**	**6.25%**	**12.5%**	**6.25%**	**p=0.01, n=472**
		**Observed frequency**	**5.93%**	**11.86%**	**4.24%**	**18.01%**	**29.45%**	**7.42%**	**8.47%**	**11.44%**	**3.18%**	

Supplementary Table 4. Polygenic *Brca2*$^{\Delta 27/\Delta 27}$ *+Rad51c*$^{dah/dah}$ mice are born at sub-Mendelian frequency. Observed genotypes of *Brca2*$^{\Delta 27/\Delta 27}$ *+Rad51c*$^{dah/dah}$ mice was determined by genotyping offspring of *Brca2*$^{\Delta 27/WT}$*+Rad51c*$^{dah/dah}$ (**a**, top) and *Brca2*$^{\Delta 27/WT}$ *+Rad51c*$^{dah/WT}$ (**b**, bottom) intercross. Expected genotypes are given using Mendelian genetics. *p*- value was calculated using chi-squared test (x^2-test).

Sample ID	Gene 1	mutation type	AA	zygosity	CNA	LOH	Gene 2	Mutation type	AA	zygosity	CNA	LOH
TCGA-C8-A12T-01	BRCA1	mut	D1344H	hetero	shallowdel	yes	FANCA	CNA	n/a	n/a	DeepDel	no
TCGA-C8-A12T-01	BRCA2	mut	E3177Q	hetero	shallowdel	yes	FANCA	CNA	n/a	n/a	DeepDel	no
TCGA-AN-A046-01	BRCA2	mut	E3342K	hetero	shallowdel	yes	FANCC	mut	R555Q	hetero	diploid	no
TCGA-AN-A046-01	BRCA2	mut	E3342K	hetero	shallowdel	yes	FANCM	mut	S1808Y	hetero	diploid	no
TCGA-AN-A046-01	BRCA2	mut	K985I	hetero	shallowdel	yes	FANCC	mut	R555Q	hetero	diploid	no
TCGA-AN-A046-01	BRCA2	mut	K985I	hetero	shallowdel	yes	FANCM	mut	S1808Y	hetero	diploid	no
TCGA-A8-A085-01	BRCA2	mut	E260Sfs*15	homo	shallowdel	yes	RAD51C	cna	n/a	n/a	AMP	no
TCGA-D8-A1JP-01	BRCA2	mut	L1965Ffs*39	hetero	shallowdel	yes	FANCC	struc var	n/a	n/a	fusion	no
TCGA-D8-A27V-01	BRCA2	mut	K936Nfs*24	hetero	shallowdel	yes	FANCC	mut	X511_splice	hetero	gain	no
TCGA-D8-A27V-01	BRCA2	mut	K936Nfs*24	hetero	shallowdel	yes	FANCC	mut	P482Lfs*5	hetero	gain	no
TCGA-A2-A0ET-01	BRCA2	struc var	n/a	n/a	BR fusion	no	FANCB	mut	I418Nfs*18	hetero	diploid	no
TCGA-A8-A07I-01	BRCA2	mut	D1355Y	hetero	shallowdel	yes	RAD51C	cna	n/a	n/a	AMP	no
TCGA-D8-A27G-01	BRCA2	mut	E2175Q	hetero	diploid	no	RAD51C	cna	n/a	n/a	AMP	no
TCGA-AC-A23H-01	BRCA2	mut	D687H	hetero	diploid	no	FANCA	mut	D931H	hetero	gain	no
TCGA-AC-A23H-01	BRCA2	mut	D687H	hetero	diploid	no	FANCA	mut	E38K	homo	gain	no
TCGA-AC-A23H-01	BRCA2	mut	D1033H	hetero	diploid	no	FANCA	mut	D931H	hetero	gain	no
TCGA-AC-A23H-01	BRCA2	mut	D1033H	hetero	diploid	no	FANCA	mut	E38K	homo	gain	no
TCGA-AC-A23H-01	BRCA2	mut	D687H	hetero	diploid	no	RAD51C	mut	D141H	hetero	AMP	no
TCGA-AC-A23H-01	BRCA2	mut	D1033H	hetero	diploid	no	RAD51C	mut	D141H	hetero	AMP	no
TCGA-AC-A23H-01	BRCA2	mut	D687H	hetero	diploid	no	RAD51C	cna	n/a	n/a	AMP	no
TCGA-AC-A23H-01	BRCA2	mut	D1033H	hetero	diploid	no	RAD51C	cna	n/a	n/a	AMP	no
TCGA-AC-A23H-01	FANCA	mut	D931H	hetero	gain	no	FANCG	mut	E566K	hetero	gain	no
TCGA-AC-A23H-01	FANCA	mut	E38K	homo	gain	no	FANCG	mut	E566K	hetero	gain	no
TCGA-AC-A23H-01	FANCA	mut	D931H	hetero	gain	no	FANCI	mut	S361L	hetero	diploid	no
TCGA-AC-A23H-01	FANCA	mut	E38K	homo	gain	no	FANCI	mut	S361L	hetero	diploid	no
TCGA-EW-A1PE-01	BRCA2	mut	E1158Q	hetero	diploid	no	FANCD2	mut	E1108*	hetero	diploid	no
TCGA-EW-A1PE-01	BRCA2	mut	K1191N	hetero	diploid	no	FANCD2	mut	E1108*	hetero	diploid	no
TCGA-A2-A04P-01	BRCA2	mut	Q3206E	hetero	gain	no	FANCA	cna	n/a	n/a	AMP	no
TCGA-AN-A0XW-01	BRCA2	mut	P527T	hetero	shallowdel	yes	RAD51C	struc var	n/a	n/a	fusion	no
TCGA-AN-A0XW-01	BRCA2	mut	P527T	hetero	shallowdel	yes	RAD51C	cna	n/a	n/a	AMP	no
TCGA-A8-A06R-01	BRCA2	cna	n/a	n/a	AMP	no	FANCB	cna	n/a	n/a	DeepDel	no

TCGA-D8-A1J9-01	FANCA	mut	R52L	homo	shallowdel	yes	FANCG	cna	n/a	n/a	AMP	no
TCGA-C8-A26Y-01	FANCA	mut	D694Y	hetero	shallowdel	yes	SLX4	mut	S381G	hetero	gain	no
TCGA-A2-A04X-01	FANCM	mut	E1622del	hetero	diploid	no	RAD51C	cna	n/a	n/a	AMP	no
TCGA-D8-A1JB-01	BRCA2	cna	n/a	n/a	DeepDel	no	FANCM	mut	R1684*	hetero	diploid	no
TCGA-D8-A1JD-01	BRCA2	cna	n/a	n/a	DeepDel	no	RAD51C	mut	P21A	hetero	diploid	no
MB-0211	BRCA2	CNA	n/a	n/a	amp	no	RAD51C	CNA	n/a	n/a	amp	no
MB-0218	FANCM	CNA	n/a	n/a	amp	no	RAD51C	CNA	n/a	n/a	amp	no
MB-0291	FANCM	CNA	n/a	n/a	amp	no	RAD51C	CNA	n/a	n/a	amp	no
MB-0313	FANCA	CNA	n/a	n/a	deep del	no	FANCM	CNA	n/a	n/a	amp	no
MB-0468	BRCA2	CNA	n/a	n/a	amp	no	FANCM	CNA	n/a	n/a	amp	no
MB-0664	BRCA2	Mut	C3069Lfs*5	n/a	diploid	no	FANCA	CNA	n/a	n/a	amp	no
MB-2513	BRCA2	CNA	n/a	n/a	amp	no	FANCC	CNA	n/a	n/a	amp	no
MB-2513	BRCA2	CNA	n/a	n/a	amp	no	FANCD2	CNA	n/a	n/a	amp	no
MB-2763	FANCA	CNA	n/a	n/a	amp	no	SLX4	CNA	n/a	n/a	amp	no
MB-2847	BRCA2	CNA,Mut	T3310Nfs*17	n/a	shallow del	yes	RAD51C	CNA	n/a	n/a	amp	no
MB-3277	BRIP1	CNA	n/a	n/a	amp	no	FANCL	CNA	n/a	n/a	amp	no
MB-3396	BRCA2	CNA	n/a	n/a	deep del	no	FANCD2	Mut	R1299C	n/a	diploid	no
MB-3871	FANCA	CNA,Mut	R212G	n/a	shallow del	yes	SLX4	CNA	n/a	n/a	amp	no
MB-4331	FANCM	CNA	n/a	n/a	amp	no	RAD51C	CNA	n/a	n/a	amp	no
MB-4351	BRIP1	CNA	n/a	n/a	amp	no	FANCL	CNA	n/a	n/a	amp	no
MB-4601	BRCA2	CNA	n/a	n/a	amp	no	FANCA	CNA, Mut	R714Q	n/a	shallow del	yes
MB-4828	BRCA2	CNA	n/a	n/a	deep del	no	RAD51C	CNA	n/a	n/a	amp	no
MB-4938	BRCA2	Mut	L2654Ffs*3	n/a	diploid	no	RAD51C	CNA	n/a	n/a	amp	no
MB-4938	BRCA2	Mut	L2654Ffs*3	n/a	diploid	no	FANCD2	CNA, Mut	X330_splice	n/a	gain	no
MB-4949	FANCM	CNA	n/a	n/a	amp	no	RAD51C	CNA	n/a	n/a	amp	no
MB-5130	BRCA2	Mut	S3366Mfs*2, V3365_S3366ins	n/a	diploid	no	FANCM	CNA	n/a	n/a	amp	no
MB-5197	FANCM	CNA	n/a	n/a	amp	no	RAD51C	CNA	n/a	n/a	amp	no
MB-5275	BRCA2	Mut	G4V	n/a	diploid	no	FANCA	Mut	Q153*,E1420K	n/a	diploid	no
MB-5275	BRCA2	Mut	G4V	n/a	diploid	no	FANCD2	Mut	E369Q, S409L	n/a	diploid	no
MB-5275	FANCA	Mut	Q153*,E1420K	n/a	diploid	no	FANCD2	Mut	E369Q, S409L	n/a	diploid	no
MB-5414	FANCM	CNA	n/a	n/a	amp	no	RAD51C	CNA	n/a	n/a	amp	no
MB-5465	BRIP1	CNA	n/a	n/a	amp	no	FANCL	CNA	n/a	n/a	amp	no
MB-5518	BRCA2	CNA	n/a	n/a	amp	no	FANCD2	CNA	n/a	n/a	amp	no
MB-5540	FANCM	CNA	n/a	n/a	amp	no	RAD51C	CNA	n/a	n/a	amp	no
MB-5556	BRCA2	CNA	n/a	n/a	deep del	no	RAD51C	CNA	n/a	n/a	amp	no
MB-5593	BRCA2	Mut	V2687I	n/a	diploid	no	RAD51C	CNA	n/a	n/a	amp	no
MB-5604	BRCA1	CNA	n/a	n/a	amp	no	FANCA	CNA, Mut	R1144W	n/a	shallow del	yes
MB-5634	FANCA	CNA	n/a	n/a	amp	no	FANCG	CNA	n/a	n/a	amp	no
MB-6026	BRCA2	CNA	n/a	n/a	deep del	no	RAD51C	CNA	n/a	n/a	amp	no
MB-6100	BRCA2	Mut	E1514K	n/a	diploid	no	RAD51C	CNA	n/a	n/a	amp	no
MB-6100	BRCA2	Mut	E1514K	n/a	diploid	no	FANCF	CNA	n/a	n/a	amp	no
MB-6156	BRCA2	CNA	n/a	n/a	amp	no	RAD51C	CNA	n/a	n/a	amp	no
MB-6178	BRCA1	Mut	V452A	n/a	diploid	no	FANCA	Mut	D79Y	n/a	diploid	no
MB-6217	FANCA	Mut	A152G	n/a	diploid	no	FANCE	CNA	n/a	n/a	amp	no
MB-6329	FANCA	Mut	H292Q	n/a	diploid	no	FANCM	CNA	n/a	n/a	amp	no
MB-7028	FANCM	CNA	n/a	n/a	amp	no	RAD51C	CNA	n/a	n/a	amp	no
MB-7186	FANCA	CNA	n/a	n/a	deep del	no	SLX4	CNA	n/a	n/a	amp	no
MB-7299	FANCA	Mut	D598E	n/a	diploid	no	FANCM	CNA	n/a	n/a	amp	no
MTS-T0697	BRCA2	CNA,Mut	Q3036*	n/a	shallow del	yes	RAD51C	CNA	n/a	n/a	amp	no
MTS-T2118	BRCA2	Mut	T2662M	n/a	not available	no	FANCD2	Mut	E719G	n/a	n/a	no
MTS-T2398	BRCA1	CNA	n/a	n/a	amp	no	FANCA	Mut	H544Q, P1392S	n/a	n/a	no
MB-5556	BRCA2	CNA	n/a	n/a	deep del	no	RAD51C	CNA	n/a	n/a	amp	no

Supplementary Table 5. Individual mutation data as defined by cBioportal.com of breast cancer patient with tumors containing polygenic FANC mutations in the sixteen FANC combinations determined in Supplementary Table 1 and 2. Deep del denotes deep deletions, shallow del denotes shallow deletions, Amp denotes amplifications, CNA denotes copy number aberration, Mut denotes mutation. Hetero/homozygosity and LOH were inferred from allele frequency and CNA.

Supplementary References

1. N. G. Howlett *et al.*, Biallelic inactivation of BRCA2 in Fanconi anemia. *Science* **297**, 606-609 (2002).
2. A. R. Meetei *et al.*, A human ortholog of archaeal DNA repair protein Hef is defective in Fanconi anemia complementation group M. *Nat Genet* **37**, 958-963 (2005).
3. T. R. Singh *et al.*, Impaired FANCD2 monoubiquitination and hypersensitivity to camptothecin uniquely characterize Fanconi anemia complementation group M. *Blood* **114**, 174-180 (2009).
4. S. C. Chandrasekharappa *et al.*, Massively parallel sequencing, aCGH, and RNA-Seq technologies provide a comprehensive molecular diagnosis of Fanconi anemia. *Blood* **121**, e138-148 (2013).
5. L. Chang *et al.*, Whole exome sequencing reveals concomitant mutations of multiple FA genes in individual Fanconi anemia patients. *BMC Med Genomics* **7**, 24 (2014).
6. W. Q. Xue *et al.*, Association of BRCA2 N372H polymorphism with cancer susceptibility: a comprehensive review and meta-analysis. *Scientific reports* **4**, 6791 (2014).
7. G. Cecener *et al.*, BRCA1/2 germline mutations and their clinical importance in Turkish breast cancer patients. *Cancer Invest* **32**, 375-387 (2014).
8. L.-H. Yan, F.-H. Sun, Y. Wang, Relationship of BRCA2 N372H polymorphism and risk of cancer: a systematic meta-analysisunder PRISMA guidelines. *Int J Clin Exp Med* **11**, 10326-10335 (2018).
9. L. B. Ahlborn *et al.*, Identification of a breast cancer family double heterozygote for RAD51C and BRCA2 gene mutations. *Familial cancer* **14**, 129-133 (2015).
10. A. Meindl *et al.*, Germline mutations in breast and ovarian cancer pedigrees establish RAD51C as a human cancer susceptibility gene. *Nat Genet* **42**, 410-414 (2010).
