# Supplementary material for: Hypomorphic Brca2 and Rad51c double mutant mice display Fanconi anemia, cancer and polygenic replication stress
Source: Nat Commun. 2023 Mar 11;14:1333. doi: 10.1038/s41467-023-36933-y (PMC10008622; doi:10.1038/s41467-023-36933-y)
Supplement: Supplementary file 3 — Reporting Summary [file 41467_2023_36933_MOESM3_ESM.pdf]

nature portfolio

Corresponding author(s): Katharina Schlacher

Last updated by author(s): Feb 6, 2023

Reporting Summary

Nature Portfolio wishes to improve the reproducibility of the work that we publish. This form provides structure for consistency and transparency in reporting. For further information on Nature Portfolio policies, see our Editorial Policies and the Editorial Policy Checklist.

Statistics

For all statistical analyses, confirm that the following items are present in the figure legend, table legend, main text, or Methods section.

n/a	Confirmed	
☐	☒	The exact sample size (*n*) for each experimental group/condition, given as a discrete number and unit of measurement
☒	☐	A statement on whether measurements were taken from distinct samples or whether the same sample was measured repeatedly
☐	☒	The statistical test(s) used AND whether they are one- or two-sided<br>*Only common tests should be described solely by name; describe more complex techniques in the Methods section.*
☒	☐	A description of all covariates tested
☒	☐	A description of any assumptions or corrections, such as tests of normality and adjustment for multiple comparisons
☐	☒	A full description of the statistical parameters including central tendency (e.g. means) or other basic estimates (e.g. regression coefficient) AND variation (e.g. standard deviation) or associated estimates of uncertainty (e.g. confidence intervals)
☐	☒	For null hypothesis testing, the test statistic (e.g. *F*, *t*, *r*) with confidence intervals, effect sizes, degrees of freedom and *P* value noted<br>*Give P values as exact values whenever suitable.*
☒	☐	For Bayesian analysis, information on the choice of priors and Markov chain Monte Carlo settings
☒	☐	For hierarchical and complex designs, identification of the appropriate level for tests and full reporting of outcomes
☒	☐	Estimates of effect sizes (e.g. Cohen's *d*, Pearson's *r*), indicating how they were calculated

Our web collection on statistics for biologists contains articles on many of the points above.

Software and code

Policy information about availability of computer code

Data collection	NA
Data analysis	described in the manuscript if applicable

For manuscripts utilizing custom algorithms or software that are central to the research but not yet described in published literature, software must be made available to editors and reviewers. We strongly encourage code deposition in a community repository (e.g. GitHub). See the Nature Portfolio guidelines for submitting code & software for further information.

Data

Policy information about availability of data

All manuscripts must include a data availability statement. This statement should provide the following information, where applicable:

- Accession codes, unique identifiers, or web links for publicly available datasets
- A description of any restrictions on data availability
- For clinical datasets or third party data, please ensure that the statement adheres to our policy

All data pertaining to the results in the manuscript are available in the main text, the Supplementary materials, and Source Data file. The raw data in Supplementary Table 2 are protected and are not available due to data privacy laws. Material requests underlie compliance with institutional policies and can be made by contacting the corresponding authors.

Field-specific reporting

Please select the one below that is the best fit for your research. If you are not sure, read the appropriate sections before making your selection.

☒ Life sciences ☐ Behavioural & social sciences ☐ Ecological, evolutionary & environmental sciences

For a reference copy of the document with all sections, see nature.com/documents/nr-reporting-summary-flat.pdf

Life sciences study design

All studies must disclose on these points even when the disclosure is negative.

Sample size	described in manuscript, sample size was not predetermined, experiments performed in three biological replicas
Data exclusions	no data excluded
Replication	sample sizes described in manuscript, minimally three biological replicas where possible
Randomization	described in the manuscript when applicable, samples were allocated at random
Blinding	described in the manuscript when applicable, samples were analyzed in a blinded fashion when possible

Reporting for specific materials, systems and methods

We require information from authors about some types of materials, experimental systems and methods used in many studies. Here, indicate whether each material, system or method listed is relevant to your study. If you are not sure if a list item applies to your research, read the appropriate section before selecting a response.

Materials & experimental systems

n/a	Involved in the study	
☐	☒	Antibodies
☐	☒	Eukaryotic cell lines
☒	☐	Palaeontology and archaeology
☐	☒	Animals and other organisms
☒	☐	Human research participants
☒	☐	Clinical data
☒	☐	Dual use research of concern

Methods

n/a	Involved in the study	
☒	☐	ChIP-seq
☐	☒	Flow cytometry
☒	☐	MRI-based neuroimaging

Antibodies

Antibodies used	listed in the manuscript
Validation	validated as shown in mauscript

Eukaryotic cell lines

Policy information about cell lines

Cell line source(s)	self (generated from experimental animals)
Authentication	genotyping
Mycoplasma contamination	tested
Commonly misidentified lines (See ICLAC register)	NA

Animals and other organisms

Policy information about studies involving animals; ARRIVE guidelines recommended for reporting animal research

Laboratory animals	genetic mouse model including strain (mixed) and ages described in detail in manuscript

Wild animals	no wild animals were used
Field-collected samples	no field collected samples were used in this study
Ethics oversight	MD Anderson IACUC committee for small animal work

Note that full information on the approval of the study protocol must also be provided in the manuscript.

Flow Cytometry

Plots

Confirm that:

☒ The axis labels state the marker and fluorochrome used (e.g. CD4-FITC).

☒ The axis scales are clearly visible. Include numbers along axes only for bottom left plot of group (a 'group' is an analysis of identical markers).

☒ All plots are contour plots with outliers or pseudocolor plots.

☒ A numerical value for number of cells or percentage (with statistics) is provided.

Methodology

Sample preparation	samples were prepared using mouse thymus and bone marrow cells
Instrument	LSRFortessa X-20 Analyzer was used
Software	FlowJo 10.4.2 (FlowJo, LLC).
Cell population abundance	no sorting occured
Gating strategy	Gating strategy was performed as described in Mayle, A., Luo, M., Jeong, M. & Goodell, M. A. Flow cytometry analysis of murine hematopoietic stem cells. Cytometry A 83, 27-37, doi:10.1002/cyto.a.22093 (2013).

☒ Tick this box to confirm that a figure exemplifying the gating strategy is provided in the Supplementary Information.
